# Full Workflows for the Analysis of Gas Chromatography—Ion Mobility Spectrometry in Foodomics: Application to the Analysis of Iberian Ham Aroma

**DOI:** 10.3390/s21186156

**Published:** 2021-09-14

**Authors:** Rafael Freire, Luis Fernandez, Celia Mallafré-Muro, Andrés Martín-Gómez, Francisco Madrid-Gambin, Luciana Oliveira, Antonio Pardo, Lourdes Arce, Santiago Marco

**Affiliations:** 1 Institute for Bioengineering of Catalonia (IBEC), The Barcelona Institute of Science and Technology, 08028 Barcelona, Spain; rfreire@ibecbarcelona.eu (R.F.); cmallafre@ibecbarcelona.eu (C.M.-M.); fmadrid@becbarcelona.eu (F.M.-G.); loliveira@ibecbarcelona.eu (L.O.); santiago.marco@ub.edu (S.M.); 2 Department of Electronics and Biomedical Engineering, University of Barcelona, 08028 Barcelona, Spain; a.pardo@ub.edu; 3 Department of Analytical Chemistry, University of Córdoba, 14071 Córdoba, Spain; q02magoa@uco.es (A.M.-G.); qa1arjil@uco.es (L.A.); 4 Integrative Pharmacology and Systems Neuroscience Research Group, IMIM-Institut Hospital del Mar d’Investigacions Mèdiques, 08003 Barcelona, Spain

**Keywords:** feature extraction, food analysis, GC-IMS, PLD-DA, pre-processing

## Abstract

Gas chromatography—ion mobility spectrometry (GC-IMS) allows the fast, reliable, and inexpensive chemical composition analysis of volatile mixtures. This sensing technology has been successfully employed in food science to determine food origin, freshness and preventing alimentary fraud. However, GC-IMS data is highly dimensional, complex, and suffers from strong non-linearities, baseline problems, misalignments, peak overlaps, long peak tails, etc., all of which must be corrected to properly extract the relevant features from samples. In this work, a pipeline for signal pre-processing, followed by four different approaches for feature extraction in GC-IMS data, is presented. More precisely, these approaches consist of extracting data features from: (1) the total area of the reactant ion peak chromatogram (RIC); (2) the full RIC response; (3) the unfolded sample matrix; and (4) the ion peak volumes. The resulting pipelines for data processing were applied to a dataset consisting of two different quality class Iberian ham samples, based on their feeding regime. The ability to infer chemical information from samples was tested by comparing the classification results obtained from partial least-squares discriminant analysis (PLS-DA) and the samples’ variable importance for projection (VIP) scores. The choice of a feature extraction strategy is a trade-off between the amount of chemical information that is preserved, and the computational effort required to generate the data models.

## 1. Introduction

One of the leading indicators of food quality and freshness is aroma [1]. Volatile aroma components that are present in food not only affect the flavor but also provide a picture of its whole production process, providing a direct route for food quality control [2,3,4,5,6] as well as fraud detection [7,8,9]. The analysis of volatile constituents in food products may be carried out by two different main approaches, sensory and instrumental analyses. Sensory analysis is the evaluation of flavor and aroma by trained experts to identify and classify a pre-set of characteristics [10]. Instrumental analysis, however, refers to determining the molecules behind an aroma, using one or more chemical techniques and detectors [11,12]. The use of instrumental analyses leads to less subjective aroma evaluation with more accuracy, and richly informative techniques that elucidate the chemical features of the aroma. The comparison of human panel evaluations with objective methods has produced a very rich diversity of literature. However, the field is not without its difficulties and, on many occasions, the conclusions are problem-dependent [13,14,15,16,17,18].

Among the most popular analytical techniques, gas chromatography coupled with mass spectrometry (GC-MS) is one of the most common [19]. It combines the ability of gas chromatographs to separate complex mixtures into their chemical constituents with the capacity of mass spectrometers, which provide information that can be related to the sample’s structure. In addition to this, the GC-MS technique is sensitive, reproducible (in mass spectra), and suitable for compound identification due to the availability of spectral libraries [20,21]. Despite the many advantages offered by GC-MS, it also presents some drawbacks. GC analysis can be time-consuming, and MS requires a sophisticated vacuum system that hinders the identification of compounds in isobaric conditions and makes this technique more expensive and non-portable.

In the quest for successful food analysis, gas sensor arrays (also known as electronic noses) have been investigated for many years [22,23]. Despite the description of a large count of successful application examples in lab conditions, the translation to real field conditions has been plagued with robustness problems [24,25,26], stability problems, expensive calibration and validation; in addition, the inherent black-box characteristics of e-noses are major barriers to overcome regarding their practical use [27,28].

One alternative to GC-MS is gas chromatography–ion mobility spectrometry (GC-IMS), which is rapid, effective, and inexpensive. The compound detection level is in the order of ppb for some compounds, and most of all, it can be portable since this technique avoids the need for vacuum systems [29]. In GC-IMS, the first separation of the volatile compounds present in the sample is performed by the chromatographic column. Molecules eluting from the column interact with a reservoir of charge (reactant ions) in the ionization region, forming clusters of ions. Then, a second separation that depends on ion mobility (K) takes place: ions are, first, accelerated by an electric field and, later, slow down due to impacts with the molecules of the drift gas. As a result, ions cross the drift tube at a terminal velocity that is proportional to the electric field, and their mobility (the constant of proportionality) is ion-specific. Ion mobility depends on the ion mass and cross-section [30]. Since the length of the drift tube is constant, then the time to travel the drift tube is also ion-dependent. Finally, at the end of the drift tube, ions are detected and neutralized. The sequence of detector readings along drift time constitutes the mobility spectrum. Since a single ion mobility spectrum requires acquiring a detector signal for about 50 ms, fast evaluation of the column’s elution is possible. The typical mobility spectrum consists of a collection of peaks whose areas are associated with different ion species abundances. In particular, the area of the reactant ion peak (RIP) represents H_3_O^+^(H_2_O) *n* ions used for the ionization of neutral compounds through a charge-transfer reaction, generating the clusters of ions of the analytes. The set of spectra obtained from the same sample are arranged in a sample matrix, where one axis represents the chromatographic retention time and the other axis represents the drift time.

GC-IMS has shown its effectiveness in determining food adulteration, food classification, and freshness over the past decade [31]. For instance, Garrido-Delgado and colleagues were able to distinguish three different purity grades of olive oil using this instrument [32,33]. The same authors succeeded in characterizing a set of several markers of the purity and stability of olive oil [3]. In addition, the geographical origin [34] and adulteration [35] of olive oil have been investigated using GC-IMS. Honey and Iberian ham are both products susceptible to being adulterated, and their authenticity has been successfully tested using this technique [36,37,38]. The freshness of products such as eggs, milk, and fish has also been studied [39,40,41].

However, despite the fact that this novel hybrid technique is suitable for multiple applications, the obtained high-dimensional datasets remain challenging for data analysis. GC-IMS data commonly suffer from misalignments and baseline problems among samples [42,43,44]. Raw data has extremely high dimensionality since the retention time and the drift time are densely sampled. However, chemical information is typically sparse in the input space and using the raw data for posterior analysis using chemometrics and machine learning leads to problems due to the course of dimensionality. Additionally, without any pre-processing, samples in high-dimensional spaces often seem to be dissimilar, even if they belong to the same class. Consequently, example data is needed to generate reliable predictive models that grow exponentially with the dimensionality of the space. From a more practical perspective, manipulating high-dimensional data can lead to time-consuming, computationally intractable analysis problems. For this reason, Horsch et al. [45] systematically compared both manual and automated strategies for GC-IMS data analysis.

Two main strategies are used to handle GC-IMS data. Some authors use vendor- [26,27,28,29,30,46,47,48,49] or custom-designed software [50,51] to visualize, manually select, process and integrate important features, often available in the form of an in-house database. This first strategy is usually employed in targeted analysis when the analytes of interest are known a priori. The second strategy, normally practiced in untargeted approaches, relies on establishing pipelines for data analysis as a method to infer meaningful chemical information from samples [52]. In such a case, GC-IMS data containing thousands of signals requires intensive data pre-processing and a comprehensible (but consistent) extraction of features, including both known and unknown signatures [53]. Typical steps in GC-IMS data pre-processing are, among others, baseline correction, smoothing, denoising, 2D alignment and RIP detailing [54]. Regarding the feature extraction process, this is performed through the automatic detection of potential features, that is, peaks in sample matrices. This stage is commonly known as peak picking or peak detection. Since peaks corresponding to the same chemical signature experience tolerances in their locations across samples, clustering methods have been proposed to align the extracted features [55]. At this point, both strategies for data analysis converge. Once the relevant features are extracted from the data, an optional but often convenient dimensionality reduction stage can be performed before the sample classification. A variety of techniques for analysis have been utilized for the latter: K-nearest neighbors (KNN), support vector machine (SVM), linear discriminant analysis (LDA) and partial least-squares discriminant analysis (PLS-DA) [9]. As an example, in the past, Lourdes Arce’s lab (University of Córdoba, Spain) has used two chemometric approaches for sample classification that were compared to get the most robust model over time: (i) a non-targeted fingerprinting analysis, in which the whole GC-IMS data was processed; and (ii) a targeted approach based on manual selection and the integration of peaks of interest (markers) [56].

In this work, an automated pipeline for the signal pre-processing of GC-IMS is presented. This pipeline includes digital smoothing, baseline correction, and peak alignment stages to correct data in retention and drift time axes. Besides that, four different options for the feature extraction of GC-IMS data are explored and their performance is compared through multivariate classification results, using partial least-squares—discriminant analysis (PLS-DA) [57]. The resulting pipelines were applied to the same GC-IMS dataset, consisting of two different quality classes of Iberian ham samples. Samples were classified according to these two categories and the relevant features for sample class separation were identified.

Iberian ham is one of the most expensive cured meats in the world. It is made from the rear leg of the black Iberian pig, a species endemic to Spain and Portugal. The quality of the ham depends on the pigs’ habitat and diet. The most valuable ham comes from Iberian pigs living in the wild, roaming freely, where they feed mostly on acorns (black label). Conversely, the lowest category of Iberian ham is produced through intensive farming, where pigs are confined and fed with animal feed (white label). The price ratio between the finest and lowest categories of Iberian ham can be higher than one order of magnitude. This gives rise to the problem of alimentary fraud in the ham industry due to irregularities in product labeling. For this reason, it is commercially relevant to find fast and objective methods for the identification of ham samples according to their feeding regime [38,58].

## 2. Materials and Methods

The complete workflow for GC-IMS analysis is described in this section. This process can be roughly divided into two parts, corresponding to the separation between the sample analysis, following the methodology described by Arroyo-Manzanares et al. [38], and the data analysis realms in the workflow. The first part comprises sampling and the GC-IMS analysis protocol. The second one is related to the data analysis pipeline and includes data pre-processing and machine learning. Although this work aims to develop a general-purpose pipeline for GS-IMS data analysis, for practical purposes it has been applied to the specific case study of Iberian ham sample-quality categorization.

### 2.1. Ham Samples

A set of 57 Iberian ham samples (26 and 27 black and white labels, respectively) were obtained from 15 different food suppliers. Ham samples were sliced in portions of 1 g and placed into a 20 mL vial, closed with magnetic caps and silicone septum, and stored at 4 °C before conducting their GC-IMS analysis. The stability of the samples was checked, after which they could be stored in the fridge for 2–3 days before analysis without altering the composition of the sample.

### 2.2. Gas Chromatography—Ion Mobility Spectrometry Analysis

GC-IMS analysis was carried out with a FlavorSpec© instrument sourced from G.A.S GmbH (Dortmund, Germany). This instrument has a tritium radioactive source of energy of 6.5 KeV and a radioactive activity of 450 MBeq. The length of the drift tube was 5 cm, and the internal electric field was 400 V/cm. To minimize condensation, the drift tube was heated at 65 °C.

A syringe heated to 80 °C was used to automatically inject 100 μL (headspace) of a 1 g sample that was incubated at 70 °C for 20 min. Nitrogen in a flow of 5 mL min^−1^ was used as the carrier gas. Sample volatiles were conveyed from the injector to the polar DB-WAX capillary column of 100% polyethylene glycol (PEG), with a length of 30 m, 0.25 mm in internal diameter, and 0.50 μm in film thickness (Agilent Technologies, Santa Clara, CA, USA). Elution occurred in isothermal mode at 80° and was driven to the ionization chamber (tritium ionization source) prior to the IMS detection. Each experiment lasted 3707 s. The drift gas flow and drift tube temperature were set to 150 mL min^−1^ and 65 °C. The spectra were acquired in positive mode, and each spectrum taken was the average of 32 scans acquired every 21 ms, with a grid pulse width of 100 µs. The sampling rate for each spectrum was 150 KHz. The commercial software LAV version 2.0.0 (GAS, Dortmund, Germany) was used to acquire the data. Raw GC-IMS data were converted to CSV files and imported to MATLAB R2019b (Matworks, USA). Each of these measurements consisted of a 2-dimensional array or matrix, where a dataset that usually comprises dozens of samples defines a hyper-rectangle where x, y, and z axes correspond, respectively, to retention time, drift time, and sample number. In this data set, each sample contains 4,550,000 points.

### 2.3. Signal Pre-Processing

Raw data is extremely high-dimensional and the chemical information therein is very sparse. To reduce data dimensionality, the drift time axis was cut out to select a region of interest, and the retention time axis sampling frequency was decimated by a factor of five. That gave rise to sample matrices of 700,000 points (3500 × 1000). Several options for feature extraction will be explored in the present paper but, in all cases, signal enhancement is required to improve the signal-to-noise ratio, remove baselines, and perform peak detailing. Additionally, small instrumental drifts (e.g., due to temperature or pressure changes) in retention and drift time have to be corrected in order to ensure feature-matching across the samples [59].

**Figure 1:**
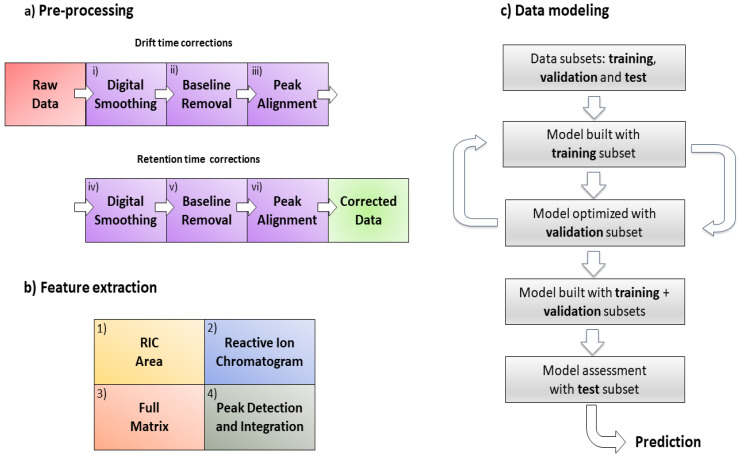
(**a**) Pre-processing workflow steps from raw data to the final matrix used; (**b**) feature extraction methods; (**c**) data modeling using PLS-DA. The first sample of the dataset was selected as a reference to correct the rest of the dataset in (**a**).

The proposed signal pre-processing pipeline to correct GC-IMS data comprises the following steps: (i) digital smoothing; (ii) baseline removal; and (iii) peak alignment on the drift time axis; after that (iv) digital smoothing; (v) baseline removal; and (vi) peak alignment on the chromatographic time axis. The pipeline for data pre-processing can be found in Figure 1a.

Digital smoothing in steps (i) and (iv) was performed using Savitzky–Golay filters [60,61], a family of digital filters that remove signal noise, reconstructing the signal by fitting successive sets of data points to low-degree polynomials (parameters: filter length, *n*; polynomial degree, (d)). Filter specifications were selected to increase the signal-to-noise ratio of the chromatograms in (i), and in the spectra in (iii), such that the height of the RIP in the drift time axis and the maximum intensity value of the RIP chromatogram were not reduced in more than 1%, respectively, for the reference sample. This gave rise to the following set of parameters: (i) *n* = 19, *d* = 2 and (iv) *n* = 27, *d* = 2. Concerning the baseline correction in (ii) and (iv), the Psalsa algorithm [44] was applied sequentially to all chromatograms, in (ii), or spectra, in (iv), in a particular sample, and for all samples. This method is based on the asymmetric least-squares (ALS) algorithm for baseline estimation [62], although it was slightly modified to reject the effects of large peaks above the baseline more easily. The Psalsa algorithm depends on three parameters: a penalty on the second derivative (λ), a penalty on the value of the baseline with respect to the value of the signal (*p*), and a third parameter, (k), that modifies substantially the value of *p* in the event of large-intensity peaks. These parameters were selected by a visual inspection of the reference sample (λ = 10^−4^, *p* = 5 × 10^−3^, and k = 200) for both drift and retention time axes.

The peak alignment in step (iii) relied on a multiplicative drift time correction for the RIP position against a reference, plus an additional linear interpolation that ensured equally sampled spectra of the same length. The reference peak was selected by averaging the RIP position within a retention time window where no compound is being eluted and for the reference sample. The computed position for the reference peak is *td* = 6.4 ms and *tr* = 190 s, where tr and td represent the retention and drift time coordinates, respectively. Peak alignment in (vi) was started by computing the total ion chromatogram (TIC) for each of the samples. Peak alignment was achieved by transforming the retention time axis of each individual sample according to the reference, using the correlation-optimized warping (COW) method [63]. In COW, the reference chromatogram of length N is divided into L segments of length I; this is performed for each sample chromatogram to be aligned. Then, the alignment between the reference and sample is performed segment by segment, allowing slight modifications to the segment lengths of the sample. Segment lengths are controlled by the so-called slack parameter, *t*. For each of the segments of a sample, the segment length is varied so that the correlation between the sample and the reference is maximized. At this point, a linear interpolation is realized to recover sample segments of the original length and sample period. Once the TIC of a sample is aligned, retention time correction is applied to each of the columns of this sample matrix. This is performed for all sample matrices. The COW method was optimized by selecting the parameters that maximized the average correlation between the reference and the rest of the samples, that is: I = 25, *t* =10 s.

### 2.4. Feature Extraction Methods

Four different methodologies for feature extraction from the GC-IMS data were compared, based on the accuracy of a downstream classifier. At this point, we would like to define the reactive ion chromatogram (RIC), a signal that can be considered as conveying similar information to the TIC in conventional chromatography. This *RIC* can be expressed as:

(1) $$RIC\left(t_{r}\right)=max\left(RIP\left(t_{r}\right)\right)-RIP\left(t_{r}\right)$$

where *RIP*(*t_r_*) is the intensity of the *RIP* peal along the retention time dimension.

The four methodologies are presented in this manuscript according to their conceptual complexity, ordered from low to high complexity levels. Their ability to extract chemical informative features from the data was studied since this factor has a direct effect on the performance of the classifier. The four extraction methods proposed are: (1) RIC areas; (2) full RIC; (3) unfolded GC-IMS preprocessed sample matrices (full matrix); and (4) peak detection and integration (Figure 1b).

In method 1, a RIC was generated for each sample, then the RIC area under the curve was calculated using the trapezoidal rule of numerical integration for each sample. That is, the GC-IMS collapses to a weighted measure of the total volatile content (WTVC), as sensed by the ion mobility detector. Here, we have to take into account that the IMS displays sensitivities that depend on the ion proton affinity for positive mode spectra. Note that in such a case, the data matrix becomes a column vector, so the approach for GC-IMS data analysis is univariate.

In method 2, the full RIC according to Equation (1) was used as a descriptor for the GC-IMS measurement. In this feature extraction method, we neglected the ion mobility spectra information and we basically treated the IMS as a zero-order detector.

The procedure for generating the data matrix in method 3 consisted of unfolding preprocessed sample matrices into row vectors. This is typically done after cutting out those spectral regions without chemical information. Thus, the data matrix was created by concatenating sample vectors by rows. In this approach we are not implementing any dimensionality reduction, so the performance of this method can be very sensitive to the curse of dimensionality. This alternative method has been employed by the Arce group in previous works [3,38,47,58].

Finally, in method 4, we consider the sparsity of the chemical information in the raw GC-IMS data, and we focus on ion peak intensities. Consequently, a peak detection and integration process was developed to obtain a peak list from every sample matrix. Using this process, the final data matrix becomes a peak table.

For this table, the algorithm uses the average matrix and finds all peaks based on two different algorithms: FastPeakFind [64] and Extrema2 [65]. The function FastPeakFind (MATLAB File Exchange, requiring Matlab’s Image Processing Toolbox) was used to select the first set of peaks. This function is a fast, robust 2D peak finder that looks for local maxima in noisy data. After peak detection, a filter was applied to select only the most intense peaks. For this filter, the peaks were sorted by intensity (ascendent sorting), and then the first derivative was applied to identify the index of the highest value, which shows the position where peak intensities depart clearly from noise. Only peaks with an index higher than 80% in the index position of this maximum occurrence were chosen. However, Extrema2 reports all the local peaks within a matrix across rows, columns, and diagonals. This second algorithm requires prior smoothing to provide good results. 

The results of both methods were merged. During the peak grouping, the function “clusterXYpoints” [66] (MATLAB File Exchange) was applied to avoid duplicated peaks. This function creates clusters of spatial points (peak positions in a matrix) according to the distance of each point to its closest neighbor. The parameters for clustering the peaks were a maximum distance of 12 points, with a maximum threshold of 6 and 15 points to the retention time and drift time, respectively. Peaks within this cluster were considered similar, and the geometric median was chosen to be the representative peak of the cluster. To correctly assign the value of the maximum intensity of a peak within a cluster, a threshold of 5 points in retention time and 10 points in drift time was selected. The highest value within each cluster and sample were identified. There are cases where the peak picking algorithm, as applied to the sample, was unable to identify a peak due to its limitations. In these cases, the intensity attributed is the intensity at the position of the representative peak in the cluster.

### 2.5. Classifier

Sample classification will be based on partial least-squares–discriminant analysis (PLS-DA), due to its ability to relate data and sample class matrices (X and Y blocks, respectively) through multivariate linear modeling with robust to low sample-to-feature ratios and feature collinearity [67,68]. It is very important to be aware that PLS-DA generally provides overoptimistic results in cross-validation [69]; for this reason, the model assessment was carried out using external validation. For all feature extraction methods described in the previous section, features were auto-scaled before training the PLS-DA data models. Additionally, robust principal component analysis (RPCA) was used to detect possible outliers [70]. Data were split into calibration (66%) and external validation subsets (34%), using the Kennard–Stone algorithm [71]. Model optimization was performed according to the flow diagram shown in Figure 1c. The complexity level of the PLS-DA models was selected by maximizing the classification rate using bootstrapping for internal cross-validation. It has been shown that bootstrapping minimizes overfitting in cross-validation, a well-known but sometimes underestimated problem in PLS-DA models [69]. The performance of the classifier was estimated in external validation (blind data) by computing the classification rate (CR) and the area under the ROC curve (AUC). The uncertainty of both estimators due to the finite sample size was estimated: CR confidence intervals were calculated using a binomial distribution, while the uncertainty of the AUC was estimated by a parametric approximation of the ROC curve, assuming a Gaussian distribution of the model numerical output per class. The significance of the classification rate at the 0.05 risk level was assessed using a permutation test [72] with 1000 repetitions.

Once the PLS-DA models were estimated, the variable importance in projection (VIP) scores for each of the different features were computed [73,74]. The variable importance in projection (VIP) scores estimate the influence of each individual variable (from the X block) in a PLS/PLS-DA model and are often used for variable ranking and selection. In a nutshell, VIP scores are computed as the weighted sum of squared PLS weights, multiplied by the amount of Y block variance, captured per latent variable and for all latent variables in the model. Note that in the previous definition, any PLS model can only have a unique VIP score vector that summarizes both the Y block and the set of latent variables. Variables with VIP scores higher than 1 can be considered influential in the model, whereas those variables lower than this value are usually excluded. The rationale for this is that the average of the squared VIP scores is 1, so the number generally chosen has a threshold value.

## 3. Results

### 3.1. Pre-Processing Results

**Figure 2:**
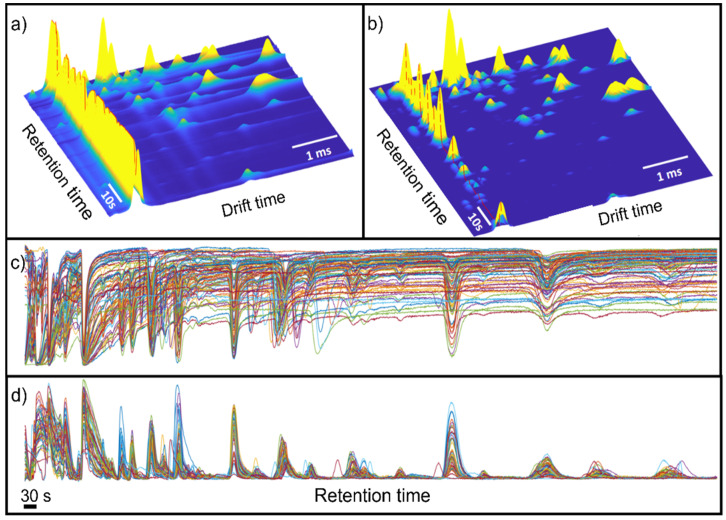
(**a**) Raw and (**b**) pre-processed GG-IMS matrix from a randomly selected black-label ham sample. For visualization purposes, the RIP region was inverted in (**b**). The dashed red line shows the RIP vector. (**c**) Raw RIP vector; (**d**) pre-processed RIP vector.

Figure 2 shows the raw data and the results after pre-processing. In this figure, it is possible to observe that the data pre-processing pipeline has corrected the imperfections of the raw data, removing the baseline and peak tailing effects that usually appear in IMS spectra. Note that the effect of baseline removal on retention time forces the RIP to have negative intensities. For visualization purposes, the RIP peak in the figure was inverted to have a positive peak.

**Figure 3:**
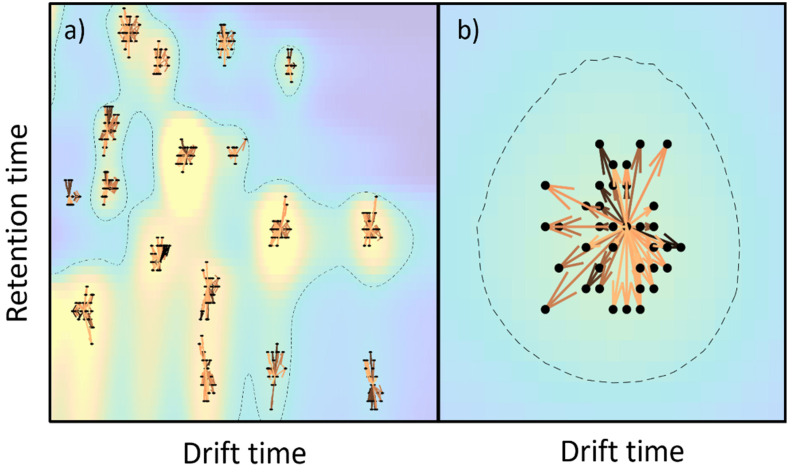
(**a**) Peak alignment across all samples (the color-coded image is the mean response across the dataset), (**b**) magnified view of peak alignment. In both subplots, arrows indicate the centroid shift, colored by time. The dashed line indicates those regions over a specific noise level.

Figure 3 shows the peak alignment and matching across the samples. First, peaks are detected for all samples, according to the methodology that was previously described. Then, peaks are clustered within a single sample and, after that, peaks are clustered across samples for peak matching. A visual inspection of the obtained results indicated a good performance for the proposed method.

### 3.2. Exploratory Analysis and Classification

**Figure 4:**
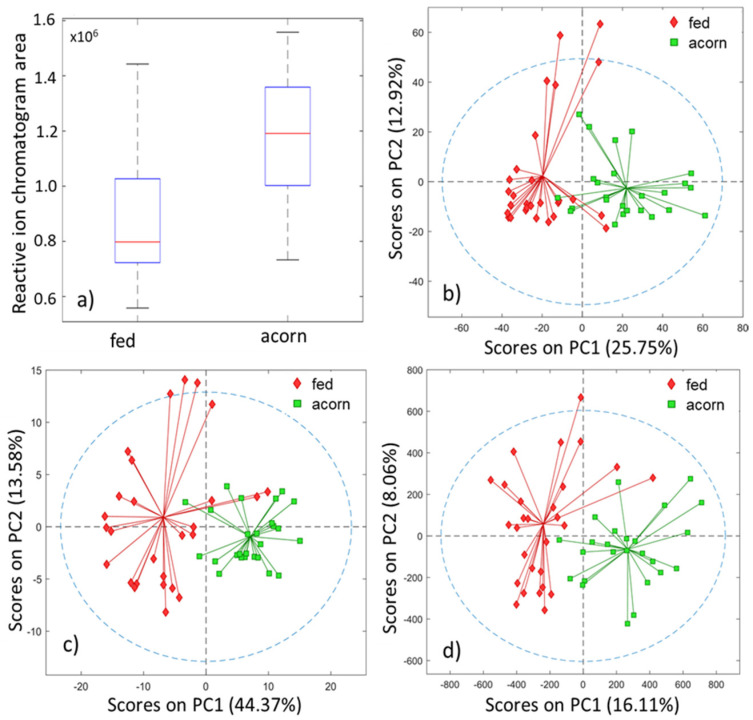
Unsupervised exploration of the four extraction methods: (**a**) boxplot for the RIC area, PCA scores plots for (**b**) reactive ion chromatogram, (**c**) full matrix, (**d**) peak table.

Evaluation of the GC-IMS data samples was performed, first in an unsupervised manner by PCA, and then in a supervised form by PLS-DA classification models, using the four proposed feature extraction methods, namely: (1) RIP areas; (2) RIC chromatograms; (3) the unfolded GC-IMS data matrices; and (4) the peak table. The boxplot for the RIC area and the PCA scores plots for the three other methods are shown in Figure 4. In all cases, unsupervised analysis already shows a good separation between both classes. Consequently, we expect good results at the classification step.

### 3.3. Classification Based on the Reactive Ion Chromatogram Area

The visual inspection of the boxplot for the RIC area shows that there is a class separation, identifiable merely by using this scalar indicator. The results seem to indicate that the total volatile organic compounds (VOC) concentration of the acorn-fed ham is greater than the one for the feed-fed ham. This would be consistent with an increasing aroma content for the former. In fact, an ANOVA shows that there is a clear mean difference between both groups.

A simple linear regression model was applied to classify those samples with a classification rate of 75% in external validation with a confidence interval of between 61 and 86% (5% risk). The area under the curve in the external validation was 0.81. The permutation test was significant, at 0.05 risk.

### 3.4. Classification Based on Reactive Ion Chromatogram

The second approach used the RIC signal as a whole instead of its total area to train and validate the prediction models.

The PCA scores plot, displayed in Figure 4b, shows a clear separation, using only the first two PCs in an unsupervised analysis. This separation was indeed confirmed and validated after performing the PLS-DA classification. The number of latent variables used for this analysis was 6 after cross-validation.

**Figure 5:**
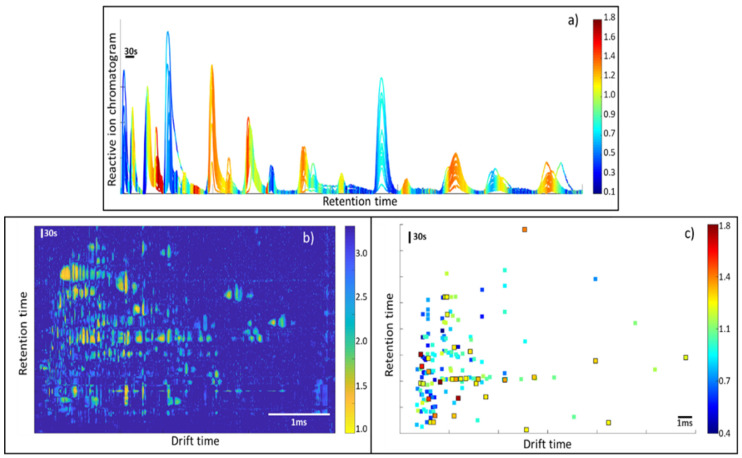
(**a**) VIPs for the reactive ion chromatogram, (**b**) VIP distribution for the full matrix (only values over VIP = 1 displayed), (**c**) VIP values for the detected peaks.

The PLS-DA model obtained an excellent classification rate of 94%, with a confidence interval ranging from 83 to 99% (5% risk). The AUC in external validation was 0.97. The probability of model insignificance from the permutation test was smaller than 10^−3^. The VIPs scores were calculated and are illustrated in Figure 5a, showing the RIC results colored according to their VIP values. It is important to realize that some very clear peaks do not have any impact on the classification, while smaller peaks can be very relevant. 

Although this approach offered a higher dimensionality than the use of the RIC area, using the whole RIC provided good classification performance and a simple data visualization of the essential features responsible for the separation (Figure 5a). This is a required feature among all quality control strategies, enabling marker monitoring and the identification of additional relevant trace compounds.

### 3.5. Classification Based on the Unfolder GC-IMS Matrices

The third approach that was evaluated was the use of the entire GC-IMS data after preprocessing. By far, this needed the highest processing demand and was the most time-consuming of all the analyses. For each sample, the pre-processed GC-IMS data first needed to be unfolded to a single vector containing 700,000 features and was then organized into a final matrix, where the rows represent the samples and the columns, the features.

Figure 3a shows a PCA scores plot with a good tendency in the separation by using two principal components. The PLS model was calculated and had a classification rate of 93%, with a confidence interval ranging from 83 to 98% (5% risk). The number of latent variables for the model was 4, obtained based on the cross-validation error. The AUC in external validation was 0.98. The probability of model insignificance from the permutation test was smaller than 10^−2^.

The VIP score obtained by this approach is a vector of 1 × 700,000 variables. To better visualize the VIPs, the vector was reshaped following the format of the original data matrix, with dimensions of 1000 × 3500. When using the whole-matrix approach, it is hard to identify the peaks responsible for the separation. The reason for this is that a single peak is composed of many VIP variables, as shown in Figure 5c. By sorting the VIPs in decreasing order, it may appear that the first 100 VIPs scores are related to only one peak region. This is because the VIP score computation does not consider correlation among variables when, in fact, these variables come from the same feature (a 2D peak).

### 3.6. Classification Based on the Peak Table

Here we describe the application of the PCA and the PLS-DA on the peak table extracted from the preprocessed GC-IMS data matrix, after the implementation of the peak detection, peak integration, and peak matching algorithms.

The PCA model was calculated and shows a good tendency in the separation using two principal components, as displayed in Figure 4d. The PLS-DA model had an excellent classification rate of 94%, with a confidence interval ranging from 84 to 99% (5% risk). The number of latent variables for the model was two, and this was obtained based on the cross-validation error plot. The AUC in the external validation was 0.96. The probability of model insignificance from the permutation test was smaller than 10^−3^. The VIP distribution is, in this case, more informative than in the previous case, since it is possible to assign a VIP value to a certain ion characterized by pair-retention time and mobility.

### 3.7. Comparison of the Results from All Approaches

The classification of the data was performed by PLS-DA on four different data matrices, each of them corresponding to a different strategy for feature extraction. Models were optimized for the best classification rate in cross-validation, but the final performance assessment was performed in external validation. Both the classification rate (CR) and the AUC were calculated with blind data. Additionally, a permutation test was performed to calculate the statistical significance of the CR being the null hypothesis, that of no-class differences. The results are summarized in Table 1. Note that all feature extraction methods demonstrated significant discrimination abilities. The inspection of the results clearly shows that the RIC area provides the poorer results while all the other three methods provide very good results. Taking into account the statistical uncertainties associated with the estimation of AUC and CR due to finite sample data, it is not possible to identify which of the three methods showed a better performance. All of them did pass the permutation test at the 0.05 risk level. The comparison among these three feature extraction methods is based on the relative advantages and disadvantages of the different approaches; these are described below.

The RIC area approach has the lowest dimensionality among all of them, compressing all the information obtained from a 2D matrix with more than one million data points into a single scalar. Within this analysis, it is impossible to infer any aspect related to chemical information, beyond an estimation of the total volatile content as weighted by the IMS sensitivity to each compound. This considerable information loss is behind the lower classification rate demonstrated. From our point of view, despite its simplicity, this method is not recommended since it hides all the chemical information from the sample analysis.

RIC chromatogram analysis is one of the most straightforward approaches to implement from the computational point of view and the algorithm development aspect. The critical step in this analysis is to correct the baseline and the peak alignment. This method obtained a considerably higher classification rate (94%). The RIC signal used to calculate the PLS-DA has 3500 data points, this being sufficiently small to perform the model calculation in real-time. Notwithstanding these advantages, the peak deconvolution remains a challenge, once this is made into a one-dimensional spectrum and thus highly dependent on the chromatographic separation. In this case, the time drift dimensionality is lost, compromising the identification of the compounds and neglecting the second-order advantage [75]. This is the method of choice if one wishes to perform fast quality control using the IMS as a detector, but it disregards the ion mobility spectra information.

The unfolded GC-IMS sample matrix approach is, by far, the most computationally intensive method to implement. The final matrix to perform the data analysis contains dozens of millions of data points, and it can take hours to evaluate the goodness of fit of a model because the cross-validation and permutation calculations are very long, due to the data’s high dimensionality. Besides, the pre-processing steps also take considerable time and need to be performed in both retention time and drift time dimensionalities. It is worth mentioning that building these corrections and data models requires a computer with enough memory to accommodate all these large matrices. This method is advantageous for identifying the compounds that are responsible for the separation, especially if they are in small concentrations for which the peak detection algorithm may not be sufficiently sensitive. Usually, a peak from a minor metabolite is challenging to detect, due to its proximity to the noise level. Its main drawback is that this vast number of data variables can lead to model overfitting, and this makes feature interpretation very challenging. Many of the variables that are important for model separation can be related to the noise. Because of this factor, a routine must be created for correct interpretation and VIP peak detection. It is also the method that is most sensitive to misalignments, especially in retention time. If misalignments are class-dependent and are not properly corrected, the algorithm may be unreliable.

Peak-based feature extraction is one of the most challenging approaches to implement from the algorithmic point of view. Beyond common preprocessing, the algorithm’s performance is very dependent on the peak detection capacities. The algorithm of peak detection should be able to correctly find and assign all peaks from all the samples, even if this peak suffers a drift or/and is found at a smaller intensity. In this algorithm, peak-matching across samples is critical to achieving a consistent peak table for the subsequent machine-learning process. Peak matching is hindered by instrumental shifts. Despite that, this approach is one of the best to implement in the sense of sample classification and the chemical information retrieved. The final matrix is tiny, having only 189 features, which is less sensitive to overfitting and decreases the computational cost during model-building. The chemical information retrieved is straightforward, where each peak is related to a single ion characterizing a specific drift time and time retention pair.

### 3.8. Comparison among the Multivariate Approaches

In this section, we aim to analyze the feature ranking obtained from VIP values across the different feature-extraction methods. The peak table strategy was used as a base to compare the methods. Retention time and drift time position were used to identify the analytes across the models. The peaks and the matrix analysis have both axes, namely retention time and drift time, enabling a direct comparison. For the full RIC, we only used the retention time to check if there was a correspondence between the results. That is, we assumed that a peak in the RIC is linked with the peak ion that shares the same retention time and has the highest intensity.

**Figure 6:**
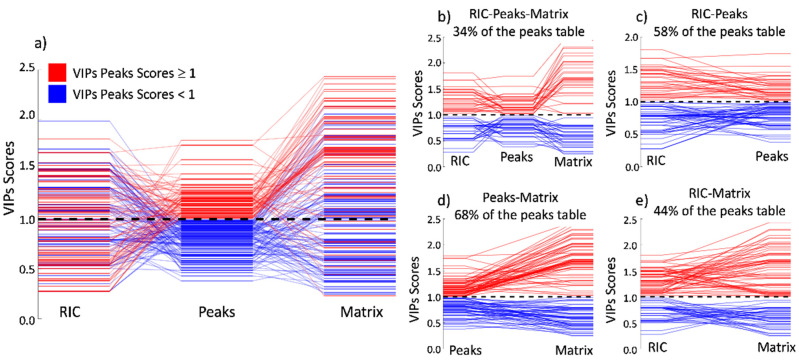
Permutation of the VIP score positions compared to the peaks model. (**a**) Comparison among the three approaches; (**b**–**e**) shows only the VIPs scores ratio that maintains its importance in the peer comparison. The higher matching occurs between the peak table and the full matrix, where 58% of the peaks keep their relative importance.

To easily visualize and compare the outcome of the VIP scores among all three methods, we created Figure 6a. In this figure, the VIPs for the peaks model were plotted in the center of the image and binary-colored according to importance. Red represents the VIPs scores that are more important for the class separation, and blue represents the VIPs scores that have no importance. In each column, you may observe that the VIP values for the described model and the diagonal lines show the permutation of the respective VIP index among the techniques.

In Figure 6a and following the diagonal lines starting from the peak’s strategy to the matrix strategy, it is possible to observe that the main tendency in the position of the importance of the VIP scores remains constant. This is as expected since both methods refer to the same peaks, proving that the separation is due to the chemical composition of the sample and is not an artifact of the data processing or any other factor. The bigger amplitude of the VIP scores in the matrix model is due to the higher number of features inducing a bigger span. Figure 6d shows precisely that 68% of the total peaks kept the same importance, whether low (blue) or high (red), between this paired comparison. In addition, the absolute values for the high importance VIPs (red) tend to increase, having higher values in the matrix model when compared to the peaks model. Conversely, the absolute values for the low importance VIPs (blue) tend to decrease, having smaller values in the matrix approach when compared to the peaks approach.

When comparing the VIPs from the peaks model to the RIC model in Figure 6a, the diagonal lines, which show the index permutation according to the peaks model, tend to scramble without showing a visual tendency as observed in the previous case. This is because the loss of the drift time dimension causes the peaks to overlap easily in the retention time dimension. In this situation, a peak that has a high VIP score in the peaks model can be significantly reduced if this peak is convoluted with other(s) peaks that are not important to the model separation. Although having this visual disorder, 58% of the peaks maintain their importance, as shown in Figure 6c. 

## 4. Discussion

Signal preprocessing and feature extraction for GC-IMS data analysis are key elements. In this paper, we have proposed a full signal preprocessing pipeline (Figure 1a), that improved both the quality of the data and the posterior performance of the peak detection and peak clustering algorithms. The use of the PLS-DA classifier and the associated feature ranking based on PLS-DA VIPs allowed us to translate the class separation to relevant chemical signatures, identified by their retention time and mobility. However, analyte identification remains a challenge due to the limited resolution and the limited size of electrical mobility databases.

In this study, different approaches for feature extraction in GC-IMS data in combination with PLS-DA classifiers are used to classify Iberian ham samples according to the pigs’ feeding regime. All the methods, except the total area model of the RIC, were able to separate the two classes of ham with high accuracy, providing an indication of the technical feasibility of this approach for quality control and fraud detection. The method of choice will be highly dependent on which information the user would like to retrieve from the analysis, as well as the expertise of the data analyst. For a simple quality-control analysis, in which the goal is only to classify the samples, the RIC approach should be considered the method of choice. This method requires low processing power, and it is the easiest method to implement as a routine analysis. However, the user needs to bear in mind that it is complicated to identify the compounds that are responsible for classification since the compounds are separated only in the chromatographic dimension and there is a great deal of coelution.

On the other hand, if the purpose of the classification is to understand the chemical nature of the samples, the unfolded sample matrix approach, followed by VIP score clustering or the peak table approach, should be preferred. In both strategies, each peak represents a substance, interpreting the features directly correlated with the compounds. Lastly, regardless of the choice of model, all the given models must be adequately validated and statistically assessed in the future.

## Figures and Tables

**Table 1:** Comparison of the performance for the different feature extraction methods. * AUC confidence Interval calculated using a parametric Gaussian approximation for the predictive output. ** A 95% confidence interval has been computed according to the binomial distribution, taking into account the finite number of samples used for the estimation of the proportion.

	Area of the RIC	RIC	Full Matrix	Peak Table
AUC in Cross-Validation	0.79	0.99	0.96	0.97
CR in Cross-Validation	77%	98%	95%	95%
AUC in External Validation	0.81	0.97	0.98	0.96
95% Conf. Interval for AUC *	0.65–0.91	0.88–0.99	0.89–0.99	0.88–0.99
CR in External Validation	75%	94%	93%	94%
95% Conf. Interval for CR **	61–86%	83–99%	83–98%	84–99%
Computational Cost	Very Low	Very Low	High	Moderate
Implementation Difficulty	Easy	Easy	Moderate	Difficult
Chemical Information	None	Moderate	Very High	High
Number of Features	1	3500	700,000	189
